# The HEMS Medical Crew Survey

**DOI:** 10.1186/1757-7241-23-S2-A28

**Published:** 2015-09-11

**Authors:** Kristen Rasmussen, Stephen JM Sollid

**Affiliations:** 1 Research Department, Norwegian Air Ambulance Foundation, Drøbak, Norway; 2 Network for Medical Sciences, University of Stavanger, Stavanger, Norway; 3 Møre and Romsdal Health Trust, Ålesund, Norway

## Background

Crew configuration in helicopter emergency medical service (HEMS) is a long debated topic. Different systems have seemingly good arguments for their choice of crew concept, but there is no solid scientific evidence available to support the benefit of any crew concept, especially in terms of medical benefit for the patient. To evaluate the rationale behind different crew compositions we invited international HEMS systems to participate in a survey to document crew concepts, crew competence and perceived benefit or disadvantage of the different crew concepts; the HEMS medical crew survey. We here present preliminary data from the survey.

## Method

Medical directors of HEMS-services in Europe, North America, Australia, New Zealand and Japan were invited to complete a web based questionnaire (SurveyXact^TM^) with five parts: “Basic information of your service”, “Mission data from 2013”, “Regular crew”, “Additional personnel by demand” and “Evaluation of crew configuration”. The survey was open between June 1st and October 15^th^, 2014. All respondents were blinded to the researcher. The study was approved by the Data Protection Official in Norway and exempted from ethical approval.

## Results

The survey received 111 submissions. Forty-four submissions did not contain sufficient data regarding crew and were excluded. The remaining 67 submissions had the following geographical distribution: Australia 3, Austria 1, Czech Republic 7, Finland 9, France 2, Greece 1, Hungary 1, Ireland 1, Japan 1, Netherlands 4, Norway 5, Poland 1, Spain 4, Sweden 3, Switzerland 2, United Arab Emirates 1, United Kingdom 4 and USA 17. Of all systems 73.1% had a 3-crew system, 23.9% a 4-crew system and 3.0% had a regular crew with 5 or 6 on board. Most services operated single pilot (85.1%), whereas the rest operated with two pilots (14.9%). In six (9.0%) services other non-medical crew such as hoist operator, mechanic or rescuer were part of the regular crew.

Medical staffing in HEMS was reported as a combination of two or more of the following categories: physician (73.1%), nurse (52.2%), emergency medical technician (EMT)/paramedic (40.3%), hems crew member (HCM) (38.8%) and respiratory therapist (RT) (6.0%). The HCMs in this survey are all trained as a nurse or EMT-paramedic, but in addition also act as a pilot assistant and often rescue specialist.

There was a large variety of medical staffing models reported in the survey. The three most common medical staffing models were physician and HCM (26.9%), physician and nurse (23.9%) and nurse and EMT/paramedic (16.4%).

One third (34.9%) of the respondents wanted to change the medical crew composition if allowed; 84.1% wanted a physician in the crew, 52.4% a nurse, 41.3% a HCM, 38.1% an EMT/paramedic and 15.9% a RT.

## Conclusion/discussion

Because we could not get access to databases of the medical directors in all the countries surveyed we cannot evaluate the response rate of our survey. Based on the systems we do know the number of HEMS systems in we know that the response rate for individual countries varies from 0 to 100%. This implies that our results cannot be fully representative of the countries surveyed. They do however provide an impression of the diversity in crew concepts and the rational behind the different crew concepts.

The survey data shows that there is no standard medical staffing, but that a combination of a physician and HCM or nurse is often found in the countries surveyed. Many HEMS systems believe that a physician must be part of the crew, but there is little consensus on the competence of the additional medical crewmember. Further studies must explore the impact of different medical crew models on patient care and patient safety to clarify which model is the best.

